# Perioperative Diagnosis and Anaesthetic Management of Idiopathic Intracranial Hypertension in Pregnancy: A Case Report

**DOI:** 10.31729/jnma.8081

**Published:** 2023-03-31

**Authors:** Prabin Subedi, Mona Sharma, Prajwala Yogi, Drishti Giri

**Affiliations:** 1 Department of Anaesthesia and Intensive Care, Kathmandu Medical College and Teaching Hospital, Sinamangal, Kathmandu, Nepal; 2 Kathmandu Medical College and Teaching Hospital, Sinamangal, Kathmandu, Nepal

**Keywords:** *case reports*, *cesarean section*, *idiopathic intracranial hypertension*, *ultrasonography*

## Abstract

Idiopathic intracranial hypertension is characterised by increased intracranial pressure of unknown aetiology. It is usually seen among obese women who are of childbearing age. With an incidence of 0.9 per 100,000 in women of childbearing age, the incidence in obese women is as high as 19.3 per 100,000. Here, we represent the case of a 31-year-old, non-obese primigravida with hypothyroidism which was diagnosed as idiopathic intracranial hypertension during pregnancy. This patient, was managed with multi-disciplinary considerations so as to avoid complications in perioperative period.

## INTRODUCTION

Idiopathic intracranial hypertension (IIH) is a condition with a benign course, characterised by increased intracranial pressure of unknown aetiology.^1^ The cerebrospinal fluid (CSF) is normal without hydrocephalus or mass lesion.^2^ With an incidence of 0.9 per 100,000 in women of childbearing age, the incidence in obese women is as high as 19.3 per 100,000.^3^ However, the rate at which intracranial hypertension occurs in pregnant women is approximately the same as in the general population.^4^ A parturient with IIH requires multidisciplinary evaluation during pregnancy and labour with special obstetric and anaesthetic considerations.^5^

## CASE REPORT

**Figure 1:**
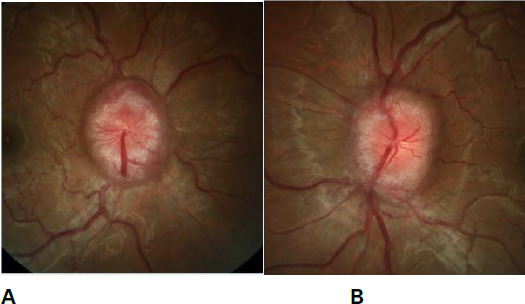
Dilatation and fundoscopic examination show bilateral disc oedema with splinter haemorrhage A) Right eye, B) Left eye.

A 31-year-old primigravida at 32 weeks of gestation with hypothyroidism visited for an Antenatal checkup Clinic. She had a history of multiple episodes of headache and had been diagnosed with migraines and treated with Tab Amitriptyline 5 years back. She doesn't give any history of smoking or alcoholism. As her symptoms had not resolved, she discontinued the medication without any medical advice. The physical examination of the patient was grossly normal. Since she had unresolved headache prior to pregnancy and further developed red flags symptoms like pulsatile tinnitus, nausea, and vomiting since 31 weeks of gestation, we advised ophthalmic evaluation to look for features of raise IIH. On ophthalmic evaluation, visual acuity was normal. Dilatation and fundoscopic examination showed bilateral disc oedema with splinter haemorrhage suggestive of papilloedema (Figure 1).

**Figure 2:**
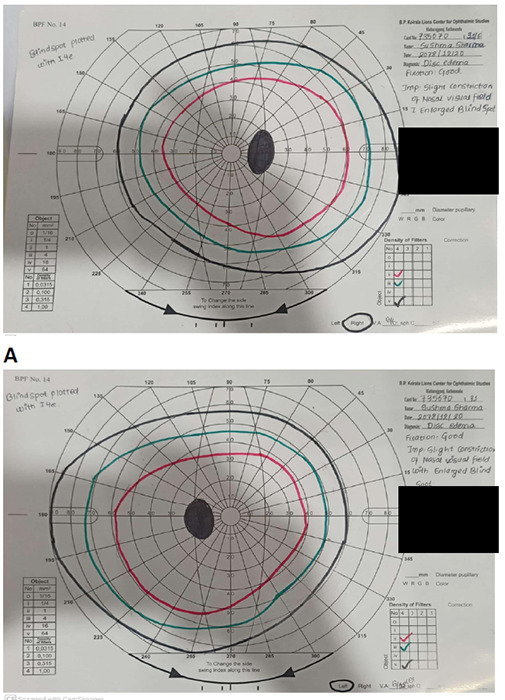
Perimetry A) Right eye shows slight constriction of nasal visual field with enlarged blind spot B) Left eye shows slight constriction of nasal visual field with enlarged blind spot.

A perimetry examination of both eyes was done. Right eye perimetry showed good fixation with slight constriction of the nasal visual field with an enlarged blind spot. Left eye perimetry demonstrated slight constriction of the nasal visual field with an enlarged blind spot (Figure 2).

**Figure 3:**
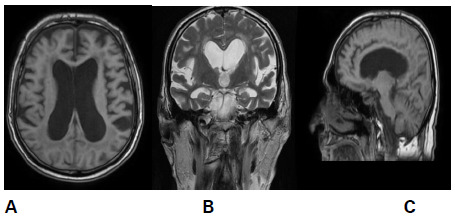
A) MRI: T1 weighted image shows dilated lateral ventricles, B) Coronal T2 weighted imaging shows acute callosal angle, C) Sagittal T1 weighted image shows upward bowing of corpus callosum.

Magnetic Resonance Imaging (MRI) was done which showed the following findings (Figure 3).

On the lumbar puncture, CSF pressure was 280 cm of H_2_O. CSF analysis was performed which was found to be normal. The patient had papilloedema on imaging. She had a normal neurological examination and CSF analysis, except for elevated CSF pressure of 28 cm CSF. So, the patient was diagnosed with Idiopathic Intracranial Hypertension at 32 weeks of gestation. She was discharged on medication, Tab Acetazolamide 250 mg BD, Tab Thyronorm 62.5 mcg OD. The patient was symptomatically better on subsequent follow-up.

After a multidisciplinary discussion, including obstetricians, neurologists, and anesthesiologists it was decided that an elective cesarean section at 38 weeks would be performed. Examination and pre-operative investigations were performed and were found to be normal. After adequate hydration and prophylaxis with a proton pump inhibitor and antiemetic, the patient underwent an elective caesarian section under spinal anaesthesia with standard American Society of Anesthesiologists monitoring. Sub-arachnoid Block was performed in the left lateral position at the L2-L3 level. A 27 G spotted needle was used for dural puncture and following which 8 mg of 0.5% Heavy Bupivacaine plus 20 mcg of Fentanyl was injected into the subarachnoid space. Adequate block level was achieved and the surgery went uneventful. Intra-operatively fluid of 1000 ml Ringer lactate was given. For multimodal pain management, Paracetamol 1000 mg and 30 mg Ketorolac was given both as injectables along with which transverse abdominis plane block was given. The postoperative period was uneventful and the patient was discharged after 3 days of hospital stay. The patient was discharged under previous medication for hypothyroidism and IIH.

## DISCUSSION

IIH can occur at any point during pregnancy although it is most commonly seen in the first half of gestation.^7^ Our patient was diagnosed with IIH at 32 weeks of gestation. The pathogenesis of IIH is still quite unclear. The proposed etiologies are: decreased CSF reabsorption, increased CSF production, cerebral oedema and an increase in blood volume.^8,9^ Age of our patient which was 31 years corresponds with the literature suggesting predominance of IIH in the women of the childbearing age group of 20-44 years.^3^ Central obesity leads to a rise in intra-abdominal pressure and subsequent rise in intrapleural pressure and cardiac filling pressure which decreases venous return from the brain and elevates intracranial venous pressure.^6^ Our case is unique because the BMI of our patient falls under the normal range.

IIH most commonly presents with headache and transient visual obscuration. Nausea, vomiting and photophobia may also be seen in association with the above-mentioned symptoms. Partial or complete visual obscuration along with visual blurring, visual loss and double vision may be seen.^7^ Rarely, patients can present with tinnitus, vertigo, CSF otorrhea and rhinorrhea.

Papilledema is the typical sign of IIH. Other signs include visual field defects with enlarged blind spots, reduced visual acuity, and reduced colour vision.^7^ Sixth nerve palsy is seen, causing diplopia which is a false localizing sign.^2^

Our patient presented with typical symptoms like headache, pulsatile tinnitus, nausea, and vomiting. Examination showed positive findings in fundoscopy and perimetry however, localizing signs including sixth nerve palsy were absent. Lumbar CSF opening pressure measured in the lateral decubitus was found to be 28 cm CSF. CSF composition was otherwise normal. Neuroimaging studies showed acute callosal angle and dilated laterally. There were no other etiologies of raised intracranial pressure. Thus, our diagnosis is compliant with Modified Dandy's criteria for IIH.^8^

The treatment of women who are pregnant and have IIH is complicated and contentious. Though the use of acetazolamide teratogenic effect seen in rodents and rabbits doesn't show any evidence of the adverse event in human pregnancy after organogenesis.^9^ So, here we advised acetazolamide after 32 weeks of gestations. Although numerous IIH review articles recommend against using acetazolamide during pregnancy, there is still insufficient clinical evidence to support this recommendation.^6,9^

Mode of labour management and delivery are also contentious. Although there is the hypothesis that uterine contraction and bearing-down force during vaginal delivery could raise CSF pressure, the rise in CSF pressure is brief, and no adverse effects have been reported from vaginal births. Because there are no published randomized controlled trials comparing the safety of neuraxial versus general anaesthesia, the decision regarding the choice of the anaesthetic technique for labour or cesarean should be individualized and discussed with the team.^5^

Additionally, there is no evidence that either mode of delivery is superior in these patients, so the recommendation is that the decision should be based on obstetric indications rather than the presence of IIH.^4^ Using either regional techniques or general anaesthesia, the primary objective is to prevent an increase in ICP.

Literature has shown that neuraxial anaesthesia, and spinal can be used as cesarean anaesthesia in patients with IIH.^10-11^ Our patient underwent LSCS under Spinal anaesthesia in the Left lateral position at the L2-L3 level. The combined use of epidural and spinal anaesthesia for cesarean section has also been reported.^11^ Spinal anaesthesia is used, especially considering that lumbar puncture is a therapeutic intervention in IIH to drain the excess CSF.^12^ Also neuraxial technique is not contraindicated in uniform raising intracranial hypertension. In patients with increased ICP resulting from space-occupying lesions, the dural puncture is contraindicated owing to the risk of uncal herniation. Increased intracranial pressure is contraindicated in epidural anaesthesia.^13^ As for IIH, herniation is prevented by uniform swelling and stiffness of the brain. The use of intrathecal opioids has considerably improved the quality of spinal anaesthesia by reducing the need for local anaesthetic and shortening the duration of motor blocking.^14^ In our patient, we add short-acting opioids as an adjuvant to enhance spinal anaesthesia. In our study acetazolamide has been used. Even when administered before the 13^th^ week of gestation, there is insufficient evidence to conclude that taking acetazolamide during human pregnancy has any negative effects.^15^ When used for multimodal analgesia to alleviate pain following a caesarean section, TAP block reduces pain, extends the duration of analgesia, and reduces the need for additional opioids.^16^ At the end of the surgery, an ultrasonography-guided transverse abdominis plane block was done for multimodal pain management to avoid the exacerbation of symptoms pertaining to raised ICP due to pain. Our multimodal analgesia strategy provided 20 hours of opioid-free analgesia postoperatively.

IIH is a rare disorder mostly affecting obese women of childbearing age. The most common presenting symptom is a headache. It is a diagnosis of exclusion in pregnant women presenting with headaches. Early diagnosis and management can help prevent transient visual obscuration and visual field defects. A multidisciplinary approach and special anaesthetic considerations are important for cesarean section or labour analgesia. The use of neuraxial anaesthesia is safe in IIH. Transverse abdominis plane block is helpful for multimodal pain management and prevention of precipitation of symptoms.
